# Deep reinforcement learning based offloading decision algorithm for vehicular edge computing

**DOI:** 10.7717/peerj-cs.1126

**Published:** 2022-10-11

**Authors:** Xi Hu, Yang Huang

**Affiliations:** Northeastern University at Qinhuangdao, Qinhuangdao, Hebei, China

**Keywords:** Vehicular edge computing, Offloading decision, Markov decision process, Deep reinforcement learning, System overhead

## Abstract

Task offloading decision is one of the core technologies of vehicular edge computing. Efficient offloading decision can not only meet the requirements of complex vehicle tasks in terms of time, energy consumption and computing performance, but also reduce the competition and consumption of network resources. Traditional distributed task offloading decision is made by vehicles based on local states and can’t maximize the resource utilization of Mobile Edge Computing (MEC) server. Moreover, the mobility of vehicles is rarely taken into consideration for simplification. This article proposes a deep reinforcement learning based task offloading decision algorithm, namely Deep Reinforcement learning based offloading decision (DROD) for Vehicular Edge Computing (VEC). In this work, the mobility of vehicles and the signal blocking commonly in VEC circumstance are considered in our optimal problem of minimizing the system overhead. For resolving the optimal problem, the DROD employs Markov decision process to model the interactions between vehicles and MEC server, and an improved deep deterministic policy gradient algorithm called NLDDPG to train the model iteratively to obtain the optimal decision. The NLDDPG takes the normalized state space as input and introduces LSTM structure into the actor-critic network for improving the efficiency of learning. Finally, two series of experiments are conducted to explore DROD. Firstly, the influences of core hyper-parameters on the performances of DROD are discussed, and the optimal values are determined. Secondly, the DROD is compared with some other baseline algorithms, and the results show that DROD is 25% better than DQN, 10% better than NLDQN and 130% better than DDDPG.

## Introduction

Since the introduction of mobile edge computing, its application scenarios are becoming more and more widespread such as autonomous driving, AR/VR, smart home, industrial internet, *etc*. As a typical service scenario of mobile edge computing, the combination of Internet of Vehicles (IoV) and Mobile Edge Computing (MEC) is called the Vehicular Edge Computing (VEC). The Vehicular Edge Computing Network not only addresses the lack of computing power in vehicles themselves, but also the problems of high latency, high energy consumption and low security in cloud computing. As a result, VEC has become a hot topic today (Mao et al., 2017). Computing task offloading is one of the core technologies in mobile edge computing. It is defined as a technology in which terminal devices hand over part or all the computing tasks to edge servers or cloud servers to solve the problem of computing resources, real-time and energy consumption of mobile devices (Flores et al., 2015). Offloading decision is one of the core issues of computing offloading technology. In the Internet of Vehicles environment, the main solution is whether the vehicular tasks need to be offloaded and where to offload (Zhang & Letaief, 2019).

At present, the offloading decision often takes delay, energy consumption, comprehensive delay and energy consumption, system utility or custom revenue as the offloading target to meet real-time needs. Luo et al. (2018) proposed to minimize the delay as the optimization goal and designed an optimization algorithm based on dynamic programming to offload the task in Luo et al. (2018). Hao et al. (2018) proposed to minimize the energy consumption as the optimization goal and designed an algorithm based on alternating iterations to offload the task in Hao et al. (2018). Han et al. (2019) proposed a joint objective optimization problem to minimize delay and energy consumption by making offloading decisions based on a heuristic algorithm. Ning et al. (2020) proposed to maximize the system utility as the goal, comprehensively consider the constraints of server storage capacity and service execution delay, and design a random algorithm based on sample average approximation. A new heuristic algorithm is proposed to transform the task offloading decision problem into a self-defined benefit maximization in Tran & Pompili (2018). The above works offload tasks from their own needs but ignore the mobility characteristics of vehicles and the characteristics of channel network transmission in the IoV. Ideally, they would not only treat the vehicle as a stationary point before and after the task is offloaded but also sees that the task transmits a good and stable signal within the range of the MEC server, but this is not the case in practice.

Considering the mobility of the vehicle and the strength of the signal in the task offloading of the Internet of Vehicles will transform the problem into an NP problem, which cannot be solved by the traditional static algorithm, so the dynamic offloading algorithm emerges as the times require, among which the algorithm based on deep reinforcement learning is particularly important striking. The offloading problem is proposed as a Markov Decision Process (MDP), and an offloading strategy based on Deep Q Network (DQN) is designed to dynamically adjust the offloading ratio to guarantee the latency and energy consumption of the system performance in Li et al. (2021). To achieve the optimal balance between the task execution delay, processing rate and energy consumption of vehicle end users, for the edge access environment of the Internet of Vehicles, Haitao et al. (2020) proposed a computing task distribution and offloading algorithm based on DQN. In task offloading, the computing power of the MEC server is not infinite. Considering the computing power of the MEC server, task offloading can be performed more effectively. Dai et al. (2021) designed a deep Q learning algorithm to solve the joint optimization problem of bandwidth, computing resource allocation and rental cost of heterogeneous servers. Wang & Wang (2021) proposed a task allocation and offloading algorithm for mobile edge computing based on deep reinforcement learning of AHP-DQN framework to solve the problems of low terminal storage capacity and diversification of network services during task offloading. Consequently, although the task offloading based on the DQN algorithm can solve the problem of dynamic offloading, the task offloading method is only limited to local computing and full offloading, ignoring various offloading types.

In Li et al. (2020) a deep deterministic policy gradient (DDPG) was proposed to optimize computational offloading for the complex computational offloading problem in the collaborative computing of heterogeneous edge computing servers (ECS). A deep deterministic policy gradient (DDPG) algorithm based on continuous action spaces is proposed in Chen & Wang (2020) to separately learn decentralized computation offloading policies for all users, aiming to make the average computational cost in a multi-user multiple-input multiple-output (MIMO) system less than the power consumption and buffering delays is minimal. The above work achieved good results in the continuous action interval task offloading decision in the non-vehicle networking field. With the increasing complexity of the environment, it has become a hot topic to apply various emerging neural networks to the field of reinforcement learning. Chen et al. (2021) proposed to apply one-dimensional convolution and long short term memory network to DDPG algorithm to solve the problem of resource allocation in Chen et al. (2021). Du et al. (2021) proposed to apply the long short term memory network to the DDPG algorithm to solve the problem of road planning and obtained good results.

Based on the above summary and analysis, we find that (1) traditional edge computing task offloading decisions are made independently by vehicles in a distributed mode, so MEC servers serve in first-in-first-service mode simply and the resource utilization efficiency is low. (2) Some characteristics of VEC should be taken into consideration, such as the high mobility of vehicles, the time-varying channel, and the signal blocking. (3) 0–1 task offloading decision is only suitable to undividable tasks, for those dividable tasks, partial offloading and the optimal offloading proportion are necessary to be considered. Therefore, this article proposes an improved DDPG based VEC-suitable central offloading decision algorithm, namely DROD. DROD can comprehensively solve the problems raised above compared with other works. The main contributions can be summarized as follows:

- Aiming to VEC environment, the mobility of vehicles, the time-varying channel and the signal blocking are considered into our optimal problem of minimizing the system overhead which is defined as the weighted average of time and energy consumption.
- A DROD algorithm based on deep reinforcement learning is proposed to obtain a full type offloading decision, that is full local computing, full offloading computing or partial offloading computing. Moreover, for partial offloading computing, the optimal offloading proportion of task can be determined by MEC server through the interactions with vehicles in its cell.
- An improved deep deterministic policy gradient algorithm, named NLDDPG, is proposed to train the neural network model and obtain the optimal decision for the optimal problem. NLDDPG improves DDPG by taking the normalized state space as input and introduces the long short term memory (LSTM) structure into the actor-critic network. The normalized state space eliminates the difference of magnitudes of different original states and speeds up training. LSTM adds history state information into Markov decision system and upgrades the training effect. Experimental simulation based on *Tensorflow* platform and verify the effectiveness of the algorithm.

The rest of article is organized as follows. In “System Model and Problem Formulation”, vehicular edge network system model and problem formulation are presented. “Deep Reinforcement learning based offloading decision (DROD) algorithm” introduces the offloading decision model based on NLDDPG algorithm. “Simulation and Result Analysis” shows the performances of NLDDPG algorithm based on simulations. Finally, “Conclusion” concludes this article.

## System model and problem formulation

### System model

**Figure 1:**
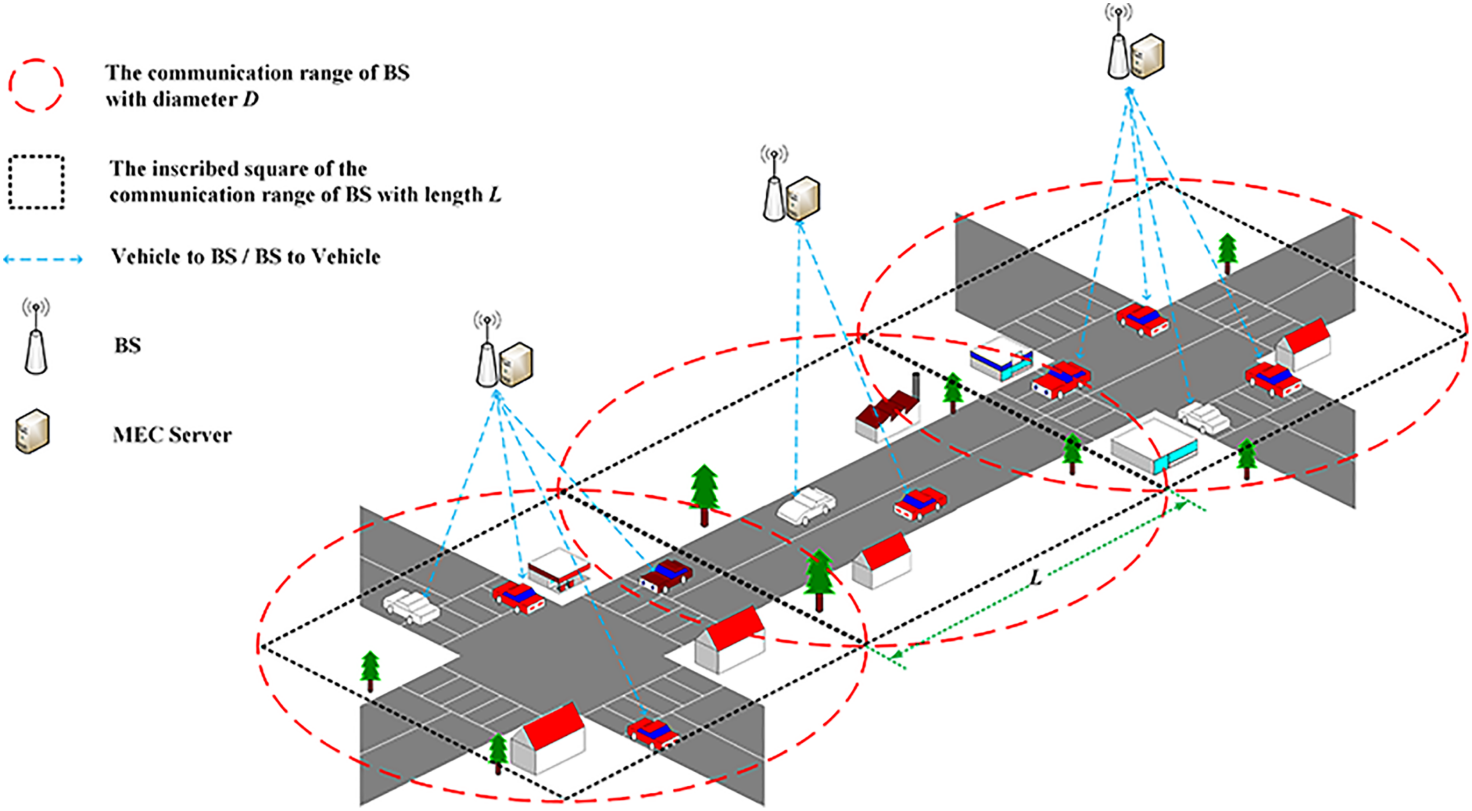
Vehicular edge computing network structure.

The vehicular edge computing network structure is shown in Fig. 1. In this structure, each base station (BS) and corresponding MEC server service the vehicles in its cell, and cooperate with each other in decentralized mode. Our work focuses on the effective offloading decision in the cell. For the decentralized vehicular decision can hardly achieve system optimality, the MEC server is selected implement offloading decision for the tasks generated by vehicles in the same cell. Let $N = \{1, 2,\ldots, n\}$ be the set of vehicles, each vehicle randomly generates a task and sends an offloading request to the BS and its corresponding MEC server performs the offloading computing. The coverage area of the BS is a circle with a diameter of *D*. Considering the overlapping coverage area of adjacent BS, a square with side length *L* inscribed in the circle is used to approximate the coverage area of the MEC server as shown in Fig. 1.

### Tasks offloading model

The task offloading ratio *x* can be used to describe the task offloading decision result in the VEC system, as shown in Eq. (1).

(1) $$\left\{ \matrix{ x = 0, & {\rm Local\, computing} \cr x = 1, & {\rm Full\, offloading}, \cr x \in (0,1), & {\rm Partial\, offloading} }\right. $$

- Local computing: Tasks are all calculated on the vehicle.
- Full offloading: All tasks are offloaded to MEC server for calculation.
- Partial offloading: A portion of the task is computed on the local vehicle, while the remainder is offloaded to MEC server for processing.

Since the connection between tasks and tasks and between tasks and MEC servers has a significant impact on task offloading decision, this article proposes a centralized decision scheme performed by MEC server. Without loss of generality, we split the decision time I of the MEC server into time slots of equal length, and within any time slot *i*, the MEC server is able to complete an offloading decision for a task request based on task characteristics and computing resources. In this way, the MEC server can choose to complete *M* task requests from a total of *S* tasks within the decision time *I* to make the VEC system optimal.

In local computing mode, the task is fully computed on vehicular Electronic Control Unit (ECU). So, the local delay *T*_*1*_ and local energy consumption *E*_*1*_ are shown in Eqs. (2) and (3).

(2) $${T_1} = \displaystyle{{\left( {1 - x} \right) \cdot {M_j}(i) \cdot s} \over {{f_{vehicle}}}},$$

(3) $${E_1} = \displaystyle{{\left( {1 - x} \right) \cdot {M_j}(i) \cdot s} \over {{f_{vehicle}}}} \cdot {P_1},$$

where, $M_j(i)$ is task data size generated by *j*-th vehicle in *i*-th time slot. *s* is the CPU cycles required to compute each bit. $f_{vehicle}$ is the ECU frequency of the vehicle. *P*_*1*_ is the unit of power consumed by the vehicle calculation. At this point, *x* is 0.

Since the location of the MEC server is fixed (Wang et al., 2019), the location of the MEC server at time t can be expressed as the coordinate ${ p} = {[p_x, p_y, H]^T}$, where *H* is the height of the BS. If the vehicular location at time *t* can be written as ${ q(t)} = {[q_x(t), q_y(t), 0]^T}$ and the driving direction does not change during the time interval $\Delta t$, the vehicular position **$ q(t+\Delta t)$** is as follows.

(4) $${q(t + { \Delta} t)} = \left[ {{q_x}(t) + v(t) \cdot \Delta t \cdot \cos \beta (t),} \right.{\left. {{q_y}(t) + v(t) \cdot \Delta t \cdot \sin \beta (t),{\rm }0} \right]^T},$$

where, $v(t)$ is the speed of the vehicle and $\beta(t)$ is the angle of the vehicle.

As shown in Eq. (5), it is worth noting that only when the vehicular coordinates **$ q(t)$** at the current moment, and the coordinate **$ q(t+\Delta t)$** of the elapsed time interval *∆t* are both within the coverage of the MEC server can it participate in the MEC server offloading computing.

(5) $${\left[ {0,0,0} \right]^T} \le {q(t),q(t + { \Delta} t)} \le {\left[ {L,L,H} \right]^T}.$$

Considering that the signal could be blocked during actual transmission, the signal blocking flag is added to differentiate the signal transmission capability. Therefore, the wireless transmission rate (Ge et al., 2020; Wang et al., 2021) is shown in Eq. (6).

(6) $${R_j}(i) = B{\log _2}\left (1 + \displaystyle{{p \cdot \alpha } \over {\left( {{\sigma ^2} + {d_j}(i){P_{loss}}} \right) \cdot {{\left\| {{q(t + { \Delta} t)} - {p}} \right\|}^2}}}\right),$$

where, *α* is the channel power gain at a reference distance 1 m. *B* is channel bandwidth. *p* is the transmission power of vehicle. $\sigma^2$ is the noise power. $P_{loss}$ is the transmission loss power. $d_j(t)$ is a flag for signal blocking $(d_j(t)=1$ indicates the presence of signal blocking).

(7) $${d_j}(t) \in \left\{ {0,1} \right\}.$$

The task is transmitted through the wireless channel with the upward transmission delay *T*_*2*_ and the upward transmission energy consumption *E*_*2*_ shown in Eqs. (8) and (9).

(8) $${T_2} = \displaystyle{{x \cdot {M_j}(i)} \over {{R_j}(i)}},$$

(9) $${E_2} = \displaystyle{{x \cdot {M_j}(i)} \over {{R_j}(i)}} \cdot {P_2},$$

where, *P*_*2*_ is the unit of power consumed by upward transmission. When *x* = 1, Eqs. (8) and (9) give the delay and energy consumption for the full offloading. The downward transmission delay and energy consumption are too small compared to the upward ones, so they are ignored (You et al., 2016).

The delay *T*_*3*_ and energy consumption *E*_*3*_ of edge computing are shown in Eqs. (10) and (11).

(10) $${T_3} = \displaystyle{{x \cdot {M_j}(i) \cdot s} \over {{f_{MEC}}}},$$

(11) $${E_3} = \displaystyle{{x \cdot {M_j}(i) \cdot s} \over {{f_{MEC}}}} \cdot {P_3},$$

where, *P*_*3*_ is the unit of power consumed by the MEC server calculation.

The time and energy consumption of partial offloading can be expressed as Eqs. (12) and (13), where $0 \lt x \lt 1$.

(12) $${T_{partial}} = \max \left\{ {{T_1},{T_2} + {T_3}} \right\},$$

(13) $${E_{partial}} = {E_1} + {E_2} + {E_3}.$$

### Problem formulation

An optimization problem is given to realize the optimal offloading decision. Its goal is minimizing the VEC system overhead through the proposed task offloading decision algorithm as described in Eq. (14).

(14) $$min \, D=\sum \limits_{i=1}^{I}C(i)\cdot{[\lambda_1(i)\cdot{(max\{T_1(i),\,T_2(i)+T_3(i) \}) + \lambda_2(i) \cdot ({E_1(i) + E_2(i)+E_3(i))]}}}$$

S.T

(14a) $$\matrix{ {{\lambda _1}(i) + {\lambda _2}(i) = 1,} & {\forall {\lambda _1}(i),{\lambda _2}(i) \in [0,1]} \cr },$$

(14b) $$max\left\{ {{T_1}(i),{T_2}(i) + {T_3}(i)} \right\} \le {T_{max}},$$

(14c) $$\matrix{ {C(i) = \sum\limits_{j = 1}^J {{C_j}(i) = 1} ,} & {{C_j}(i) \in \left\{ {0,1} \right\}} \cr },$$

(14d) $$\sum\limits_{i = 1}^I { \cdot C(i) \cdot } {M_j}(i) \le {F_{MEC}},$$

(14e) $$x(i) \in [0,1],$$

where, *D* is the system overhead. $\lambda_1(i)$ is time delay weight and $\lambda_2(i)$ is energy consumption weight. Equation (14a) indicates that the linear sum of the weights of delay and energy consumption is 1. $T_{max}$ is the maximum tolerated delay of the current task. Equation (14b) requires that the total computing delay must be no more than the maximum tolerated delay of task. $C_j(i)$ is the flag whether the *j*-th task is offloaded or not. Equation (14c) indicates that only one task can be decided by MEC server within each time slot *i*. *F*_*max*_ is the maximum computing capacity of the MEC server. Equation (14d) indicates that the total computing resources needed by tasks cannot exceed the maximum computational capacity of MEC server. The goal of this article is to optimize the variables *x*(*i*) and $C_j(i)$ so that *D* is the smallest.

### Deep reinforcement learning based offloading decision (DROD) algorithm

Different from traditional distributed offloading decision, the DROD algorithm uses central decision mode and considers task characteristics and network states synchronously. With the rich information, it can realize an effective dynamic decision for the task computing request. The key works include Markov offloading decision model and NLDDPG based optimal decision.

### Markov offloading decision model

**Figure 2:**
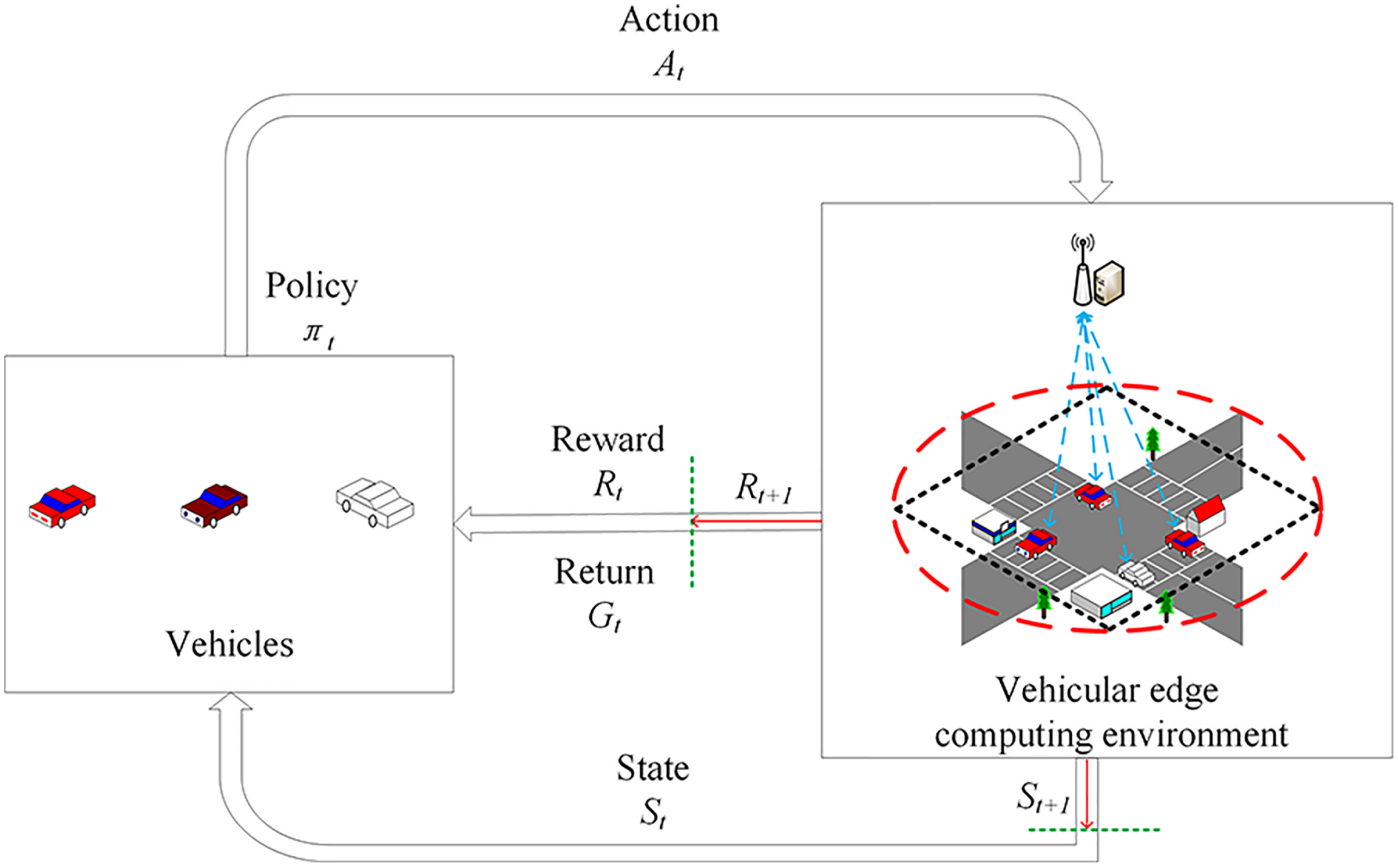
Task offloading decision model based on Markov decision process.

The Markov decision process (Wang et al., 2020) (MDP) is a mathematical model commonly used in decision making. Therefore, combining with VEC scenes, a suitable task offloading decision model is designed. This model contains a set of interactive objects, such as vehicles, MEC servers and other types of nodes, and five elements, *i.e*., state, action, policy, reward and return. As shown in Fig. 2, the interactions work in a closed loop feedback mode. The details are described as follows.

At a certain time *t*, vehicle perceives the initial state *S*_*t*_ and implements the action *A*_*t*_ according to the policy. After the action has an effect on the edge environment, it enters a new state $S_{t+1}$ and returns a reward *R*_*t*_ to the vehicular edge environment. Subsequently, the vehicle adopts a new policy $\pi _t$ based on $S_{t+1}$ and continues to interact with the environment. In this continuous interaction process, the vehicle and the environment will generate a large amount of data. The vehicle uses these generated data to adjust its own action policy, interact with the environment, generate new data and use the new data to improve its own policy. After iterating, the vehicle finally learns the optimal policy for maximizing the long term return *G*_*t*_ of the tasks.

### NLDDPG based optimal decision

Based on the above model, this article proposes an improved deep deterministic policy gradient (NLDDPG) algorithm to solve the optimization problem given by Eq. (14). NLDDPG is developed from the deep deterministic policy gradient (DDPG) (Ren et al., 2021) algorithm. The main difference is that NLDDPG enables state normalization and better extraction of task specificity, while DDPG cannot. The state normalization is an important necessary operation for NLDDPG, because it eliminates the difference in magnitude of different state values. NLDDPG introduces the LSTM structure into the actor-critic network and learns the hidden state in the partially observable Markov state through memory reasoning, which improves the learning performance of the neural network. Moreover, compared with the discrete action deep Q learning network (DQN) (Feng et al., 2021) algorithm, NLDDPG uses high dimensional continuous actions which results in better optimization, stability, and convergence.

#### Five basic elements

The deep reinforcement learning method solves the problem with five basic elements.

*Normalized state space*: In the VEC system, the state space consists of all kinds of states of MEC server and vehicle that can affect the offloading decisions.

(15) $${s_i} = ({F_{remain}}(i),{q_j}(i),{M_j}(i),{d_j}(i)),$$

where, $q_j(i),\, M_j(i)$ and $d_j(i)$ are the coordinates, task data size and block flag of *j*-th vehicle in *i*-th time slot respectively. $F_{remain}(i)$ is the remaining computing resources of the MEC server in the *i*-th time slot.

In the training process of neural network, the large value range of task data slows down the training speed. Furthermore, the differences and uncertainties of the value ranges of different task types will lead to instability and poor convergence of the entire system. So, it is necessary to normalize the value range for NLDDPG. Then the normalized state can be expressed as:

(16) $${s_i}^{\prime} = \left(\displaystyle{{{F_{remain}}(i)} \over {{F_{MEC}}}}, \displaystyle{{{q_j}(i)} \over L},\displaystyle{{{M_j}(i)} \over {{M_{sum}}}},{d_j}(i)\right),$$

where, *F*_*MEC*_ is the maximum computing resource of the MEC server and *M*_*sum*_ is the sum of the tasks participating in the offloading at the current moment.

*Action space*: According to the current state, the vehicle chooses a two-dimensional action. The action *a*_*i*_ can be expressed as:

(17) $${a_i} = (k(i),x(i)),$$

where, $k(i)$ is the vehicle selected in the *i*-th time slot and $x(i)$ is the offloading ratio of tasks in the *i*-th time slot.

*Reward*: Reward is a measure of the performance of the action selected by vehicles in the current state and it is the decisive factor for evaluating the performance of the algorithm. This article selects the action based on the merits of the reward. The goal of this article is to minimize the system overhead. According to the Eq. (14), the reward can be set as:

(18) $${r_i} = - D.$$

*Policy*: Policy can guide actions, so good policy can make vehicles produce good task offloading and improve vehicular edge computing environment. The deterministic policy *μ* adopted in this article is because the efficiency is dozens of times higher than the random policy, which greatly shortens the training time.

(19) $${a_i} = \mu \left( {{s_i}^{\prime}} \right).$$

*Return*: Return is the accumulation of rewards over time, and is the sum of all rewards on the timeline. The vehicle can learn the optimal policy through return. The goal of this article is to maximize return within each time slot.

(20) $${G_i} = \sum\limits_{l = i}^I {{\gamma ^{l - i}}{r_i}},$$

where, *γ* is the discount factor.

#### Framework outline

**Figure 3:**
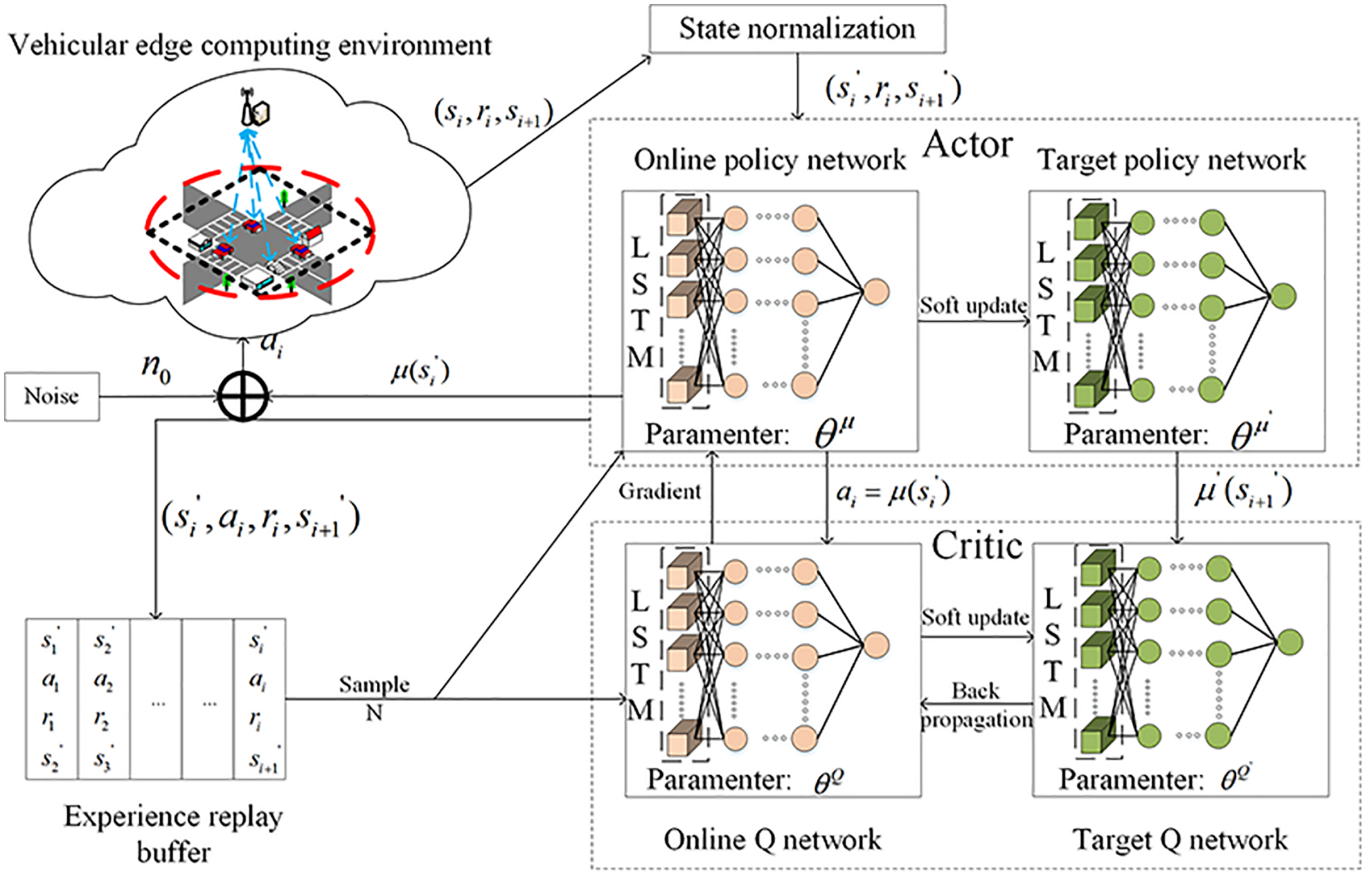
NLDDPG algorithm architecture diagram.

The architecture diagram of NLDDPG algorithm is shown in Fig. 3.

*Actor-Critic network*: The role of the actor part is to define parameterized policies and generate actions based on the observed state of the environment, while the critic part is responsible for evaluating and criticizing the current policy by processing the rewards obtained from the environment.

Due to the partial observability of environment, it is difficult for vehicles to obtain relevant information and MEC server develop successful decision when the deep reinforcement learning algorithm is trained in a complex dynamic environment. As a result, some processing of the neural network is required to produce better results. LSTM can synthesize historical and current information to handle complex state space data well. We introduce the LSTM structure into the actor-critic network, use memory reasoning to extract vehicle tasks and environmental information, and conduct effective network learning by observing the state space and analyzing data features comprehensively.

*Experience replay buffer*: The experience replay buffer mechanism refers to using a fixed replay buffer to store the previous transition $(s_i^\prime, a_i, r_i, s_{i+1}^\prime)$ and randomly selecting a fixed number of transitions from it to update the network each time. It greatly affects the training speed and final performance of the network.

*VEC*: VEC is the vehicular edge computing environment including vehicles and MEC server *etc*. In VEC, the vehicles interact with the environment and learns actor-critic network parameters through experience replay buffer to find the action with the highest rewarding.

#### NLDDPG description

The purpose of the NLDDPG algorithm is to learn the optimal policy for maximizing long term return. The input of the NLDDPG algorithm is the vehicular edge computing environment parameters such as episodes *E*, time slot *I*, actor network learning rate $\alpha_1$, critic network learning rate $\alpha_2$, discount factor $\gamma$, soft update factor $\tau$, experience replay buffer *R*, noise *n*_*0*_ and mini-batch *N*, and the output is the optimal online policy network weight $\theta^\mu$. The NLDDPG algorithm process is as follows. The first step is to randomly initialize the online policy network and online Q network weight, and copy them to the corresponding target network parameters. In the second step, the experience replay buffer is cleared and an iterative mini-batch is set. The third step is to enter the vehicular edge computing environment and start iterative process. The fourth step is to obtain the optimal weight of the actor network after the iteration is completed. The operation process of the NLDDPG algorithm (Lillicrap et al., 2015) is shown in Algorithm 1.

**Algorithm 1:** NLDDPG

Input: Vehicular edge computing environment parameters
Output: Optimal online policy network weight ${\theta ^\mu }$
1: Randomly initialize critic and actor network with online policy network weight ${\theta ^\mu }$ and online Q network weight ${\theta ^Q}$
2: Initialize target network with weights ${\theta ^\mu }^{\rm '} = {\theta ^\mu }$, ${\rm \; }{\theta ^Q}^{\rm '} = {\theta ^Q}$ and experience replay buffer *R*, mini-batch *N*
3: Enter vehicular edge computing environment
4: For episode = 1 to *E* (Max_episode) do
5: Initialize a random process for action exploration
6: Reset the parameters of the vehicular edge computing environment
7: Receive initial observation state *s*_*1*_
8: For each time slot = 1 to *T* do
9: Normalize state *s*_*i*_ to $s_i^\prime$
10: Execute action ${a_i} = \mu \left( {{s_i}^{\rm '}} \right) + {n_0}$ according to current and exploration noise *n*_*0*_
11: Calculate reward *r*_*i*_ based on (18), obtain a new state $s_{i+1}$ and Normalize state $s_{i+1}$ to $s_{i+1}^\prime$
12: Store transition $(s_i^\prime, a_i, r_i, s_{i+1}^\prime)$ in experience replay buffer *R*
If *R* is not full
Store transition in *R*
13: Else
Randomly replace any other transition in *R*
End if
14: Randomly sample *N* transitions from the replay buffer *R* as a mini-batch training data
15: Process *via* LSTM network
16: Calculate the Q value of the online Q network
${\hskip3pc}{y_i} = {r_i} + \gamma Q\left( {{s_{i + 1}}^{\prime},\mu \left( {{s_{i + 1}}^{\prime}} \right){\rm |}{\theta ^Q}} \right)$
17: Gradient back propagation update weight of online Q network by using strategy gradient
${\hskip3pc}L\left( {{\theta ^Q}} \right) = \displaystyle{1 \over N}\mathop \sum \limits_{j = 1}^N {\left( {{y_i} - Q\left( {{s_i}^{\prime},{a_i}{\rm |}{\theta ^Q}} \right)} \right)^2}$
18: Gradient back propagation update the weight of online policy network
${\hskip3pc}{\nabla _{{\theta ^\mu }}}J \approx \displaystyle{1 \over N}\mathop \sum \limits_{j = 1}^N \left[ {{{\left. {{\nabla _a}Q\left( {s,a\left| {{\theta ^\mu }} \right.} \right)} \right|}_{s = {s_i}^{\prime},a = \mu \left( {{s_i}^{\prime}} \right)\left| {{\theta ^\mu }} \right.}}{{\left. {{\nabla _{{\theta ^\mu }}}\mu \left( {s{\rm |}{\theta ^\mu }} \right)} \right|}_{s = {s_i}^{\prime}}}} \right]$
19: Soft update target network
${\hskip3pc}{\theta ^Q}^{\prime} = \tau {\theta ^Q} + \left( {1 - \tau } \right){\theta ^Q}^{\prime}$
$\hskip3pc{\theta ^\mu }^{\prime} = \tau {\theta ^\mu } + \left( {1 - \tau } \right){\theta ^\mu }^{\prime}$
20: End for
21: End for

where, $Q\left( {s,a\left| {{\theta ^Q}} \right.} \right)$ and $Q\left( {s,a\left| {{\theta ^\mu }} \right.} \right)$ are the state-action value functions of the critic and actor network respectively.

### DROD algorithm

DROD is a task offloading decision algorithm using NLDDPG, and the details are shown in Algorithm 2.

**Algorithm 2:** DROD

Input: Optimized weights of online policy network ${\theta ^\mu }$, Number of time slots *T*, Initial state *s*_*1*_
Output: Optimal offloading decision (minimum system overhead, optimal offloading vehicle and optimal offloading ratio)
1: Initialize the vehicular edge environment and the vehicle generates task requests
2: If task is divisible
3: If coordinates are within MEC server range when vehicle requests the task and receives result
4: Perform NLDDPG algorithm to obtain optimized weights of online policy network ${\theta ^\mu }$
5: Get the optimal offloading decision
6: Else
7: Enter step (9)
End if
8: Else
9: Calculate the system overhead of local computing and full offloading
10: Compare and get the optimal offloading decision
11: End if

## Simulation and result analysis

### Simulation environment and parameter setting

We have simulated a MEC environment with Python. In this environment, there are one MEC server and ten vehicles. The MEC server is allocated besides road, and all vehicles move along the road with a random chosen angle in the communication region of the MEC server. In the simulation, vehicles generate task and send requests to the MEC server. When the MEC server receives these requests, it makes an offloading decision according DROD algorithm. DROD was tested on *Tensorflow* platform with Python programming. All simulation parameters used are shown in Table 1. It should be noted that the specific numerical parameter values of VEC network used in the simulation are used to verify the effectiveness of the algorithm. The parameter settings only need to be reasonable in accordance with the actual environment. Referring to the works (Li et al., 2018; Tran & Pompili, 2018; Wang et al., 2019; Wang, Wang & Liu, 2021), the similar values are set in our simulations.

**Table 1:** Related parameters of NLDDPG.

Parameters	Values
Neural network	LSTM
Layers	2
Neurons	100, 10
Batch size	64
Learning rate	6e–7
Soft update factor	0.01
Episode	600
Maximum MEC calculation capability	10 Gbit
Vehicular speed	10 m/s
vehicles	10
The weight of delay	0.5
The weight of energy consumption	0.5
Computing frequency of the vehicle	1.2 GHz
Computing frequency of the MEC server	5 GHz
The length of the inscribed square *L*	1,000 m
Task size	[1,2], [20,30], [200, 250] Mb
The CPU cycles needed to compute a bit	1,000 cycles/bit

### Results and analysis

#### Selection of hyper-parameters

In the NLDDPG algorithm, the setting of hyper-parameters is very important. Different hyper-parameters will affect the optimization, convergence and stability of the algorithm. Therefore, this article has done relevant experiments to determine the optimal hyper-parameters used in the algorithm.

**Figure 4:**
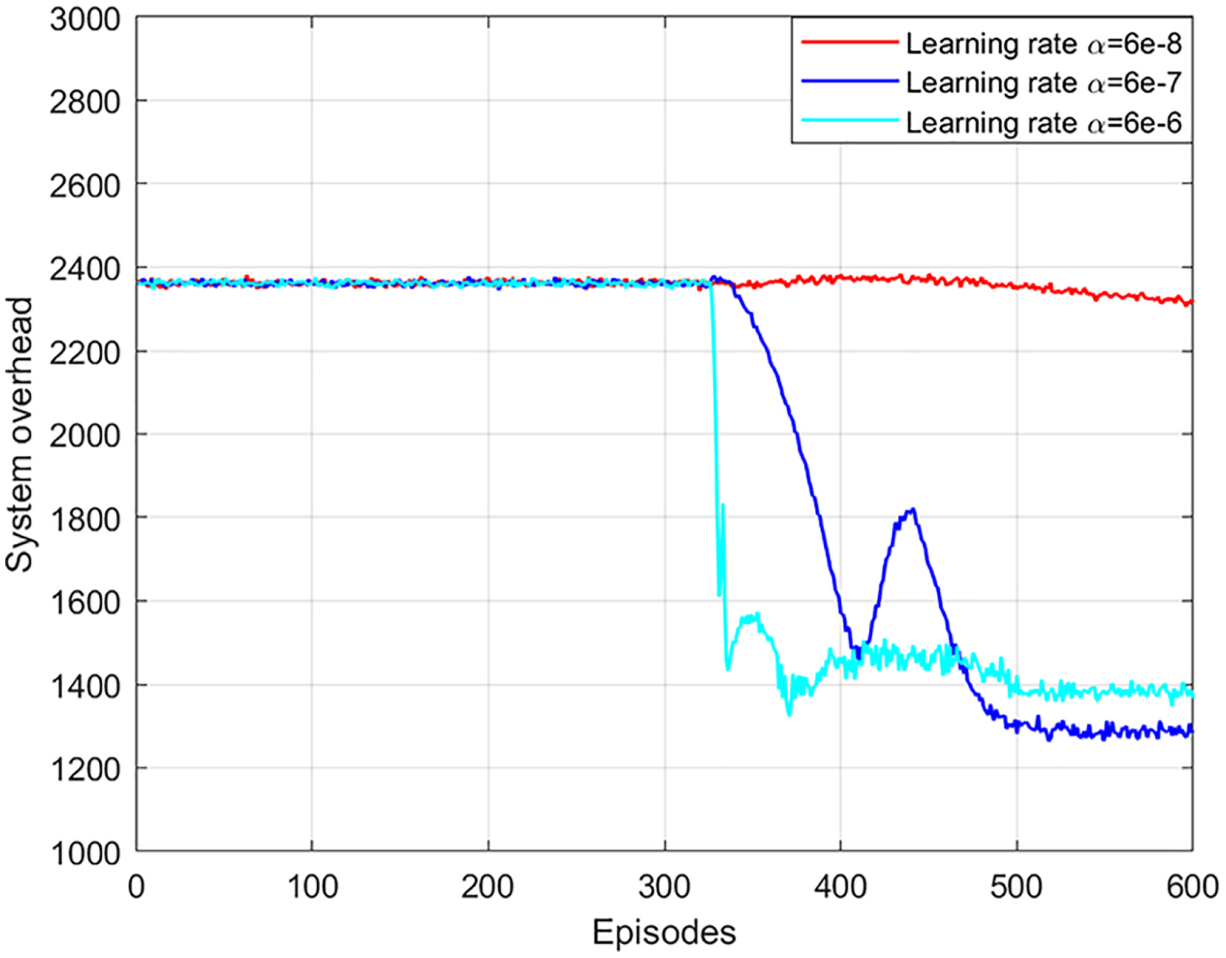
System overhead *vs* network learning rate.

The network learning rate affects the training and updating of the neural network in the NLDDPG algorithm. A large network learning rate leads to poor optimization and stability of results, while a small network learning rate leads to poor optimization and slow convergence. In general, the actor network and critic network are symmetric, so the learning rate is the same. As shown in Fig. 4, when the learning rate of the network is 6e−6, the system overhead decreases as the number of iterations increases. But the optimal solution here is not as good as the learning rate of 6e−7. The reason for this is that a large learning rate causes both the critic and actor networks to have a large update rate, while the best solution only requires minimal updates. When the learning rate of the network is 6e−7, the system overhead can obtain a convergent and stable optimal solution. Although the local optimal solution is obtained at 410 episodes, the global optimal solution can still be obtained as the number of iterations increases. When the learning rate of the network is 6e−8, the system overhead cannot obtain the optimal solution, which is due to the fact that a lower learning rate leads to a slower neural network update rate, which requires more iteration sets to converge. Consequently, the optimal network learning rate in this article is 6e−7. In addition, it can be seen from the figure that after 300 episodes, the system overhead has a large change. This is because the experience replay buffer is full at this time, and the neural network learns to have enough useful information for training.

**Figure 5:**
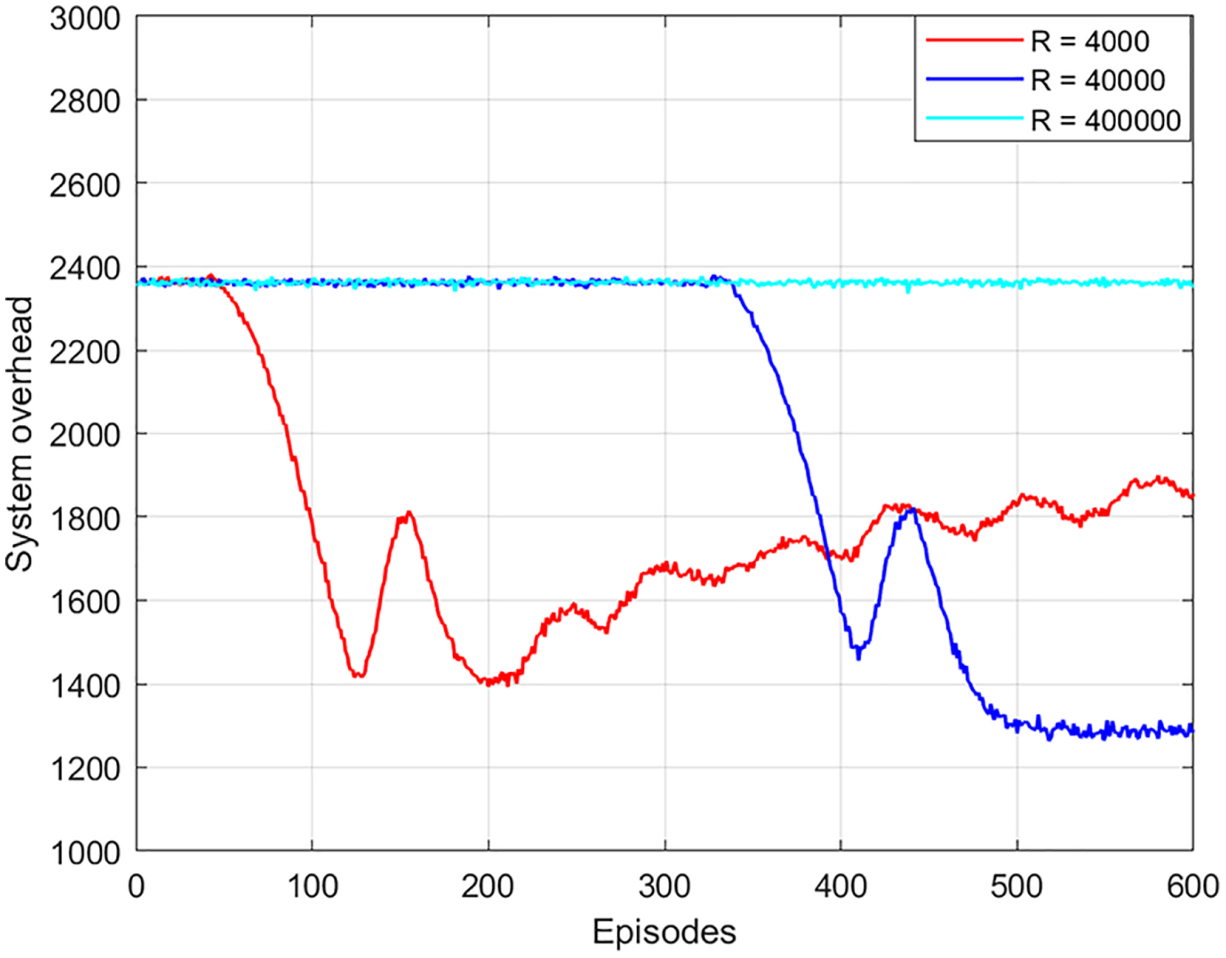
System overhead *vs* experience replay buffer.

The experience replay buffer affects the training time, optimality, and convergence of the algorithm. As shown in Fig. 5, the local optimal solution is obtained in 200 episodes or an experience replay of 4,000. However, the global optimal solution cannot be obtained. The reason is that the experience replay is too small, which will affect the feature information of the extracted data and cannot learn the optimal strategy. When the experience replay is 40,000, the global optimal solution is obtained after 490 episodes. When the experience replay is 400,000, the optimal solution cannot be obtained. This is because the buffer is too large and the data cannot be updated and thus the optimal solution cannot be obtained. In addition, the training time of the algorithm with the experience replay buffer of 40,000 times is 2.5 times faster than that of the experience replay buffer of 400,000 times. As a result, this article chooses the best experience replay buffer as 40,000.

#### Performance comparison and analysis

**Figure 6:**
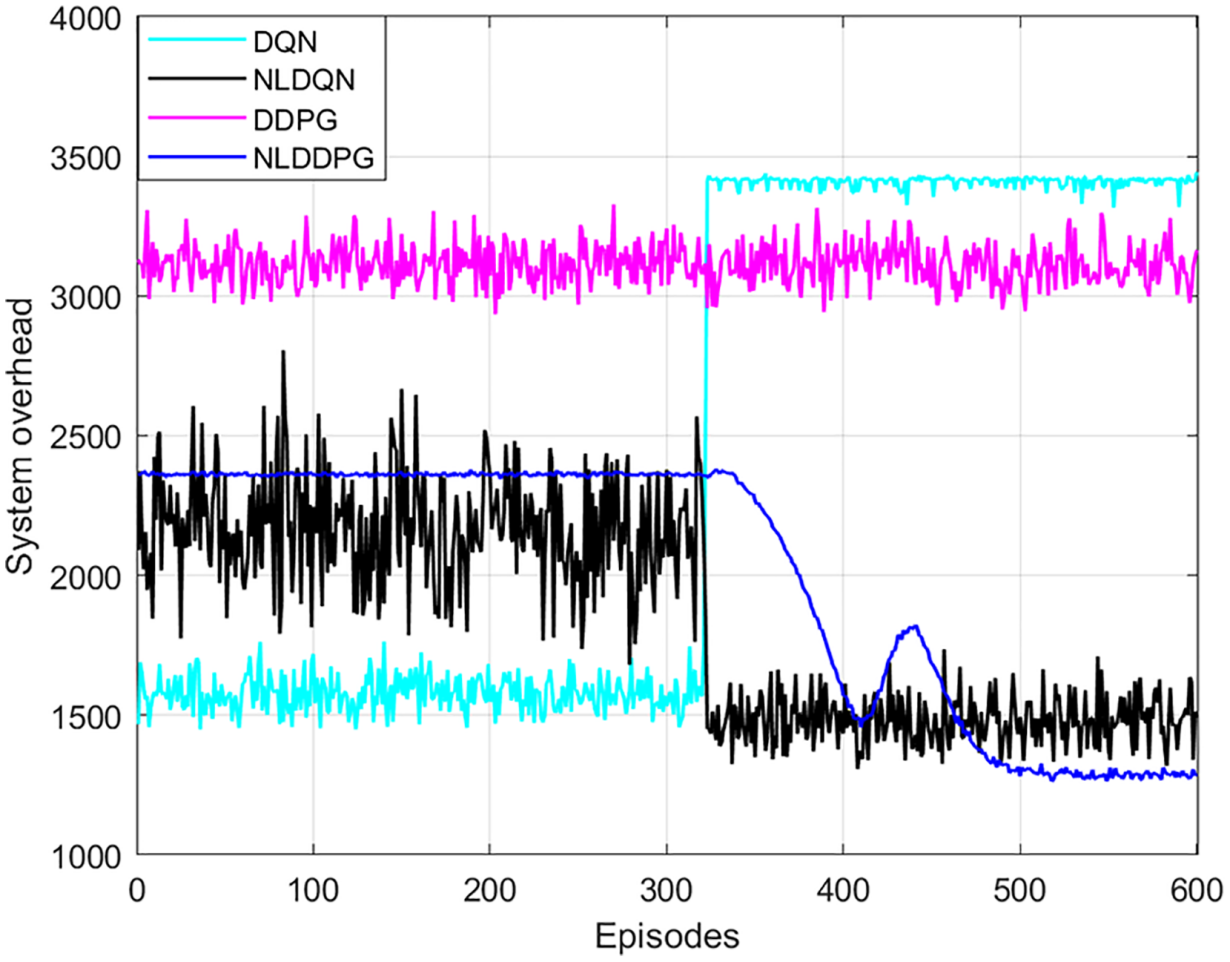
System overhead of different algorithm under different episodes.

To verify the performance of the NLDDPG algorithm, the DQN algorithm, the normalized DQN algorithm with LSTM network (NLDQN) and the DDPG algorithm were used for comparison experiments. Figure 6 shows that as the number of iterations increases, the NLDDPG algorithm has the lowest system overhead and the best stability after 420 episodes. In contrast, the DQN algorithm rises in the other direction after 320 episodes, since the DQN’s randomization strategy has the opposite impact as the one we want. The NLDQN algorithm can get the optimal solution after 320 episodes. However, the stability and convergence of the DLDQN algorithm are poor, and the optimal result cannot be found. This is because the discrete space of DLDQN cannot precisely determine the optimal offloading strategy. The DDPG algorithm varies somewhat as the number of episodes increases, however it has a weak optimality finding algorithm. This is due to the complex vehicular edge environment, such as the large number of tasks and vehicles, which exceeds the ability of the neural network and affects the optimization results. Therefore, adding LSTM network to the neural network to extract features in the environment and normalizing the state space can improve the effect. As a result, the NLDDPG algorithm outperforms the DQN algorithm, NLDQN algorithm and the DDPG algorithm in terms of optimality and stability.

**Figure 7:**
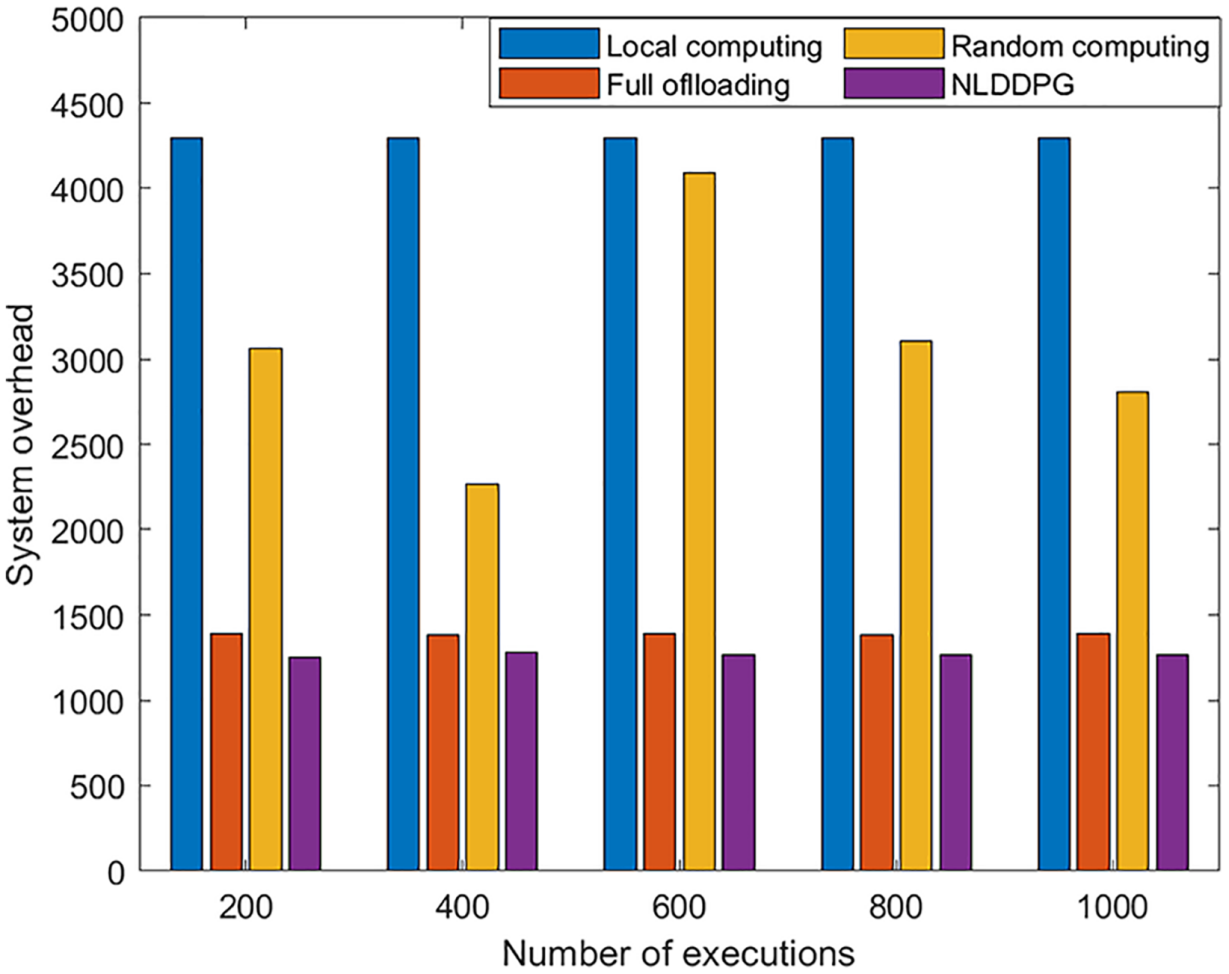
System overhead value of different offloading strategy under different executions.

To further examine the performance of the NLDDPG algorithm, it is compared to local computing, full offloading, and random computing. Random computing refers to the offloading method in which the offloading ratio is randomly selected within [0,1]. In order to verify the stability and effectiveness of the algorithm, Fig. 7 is used to represent the relationship between the number of algorithm executions and the system overhead. As shown in the Fig. 7, the system overhead of local computing, full offloading and NLDDPG algorithm remains stable as the number of executions increases, but the system overhead of NLDDPG algorithm remains the lowest. This is because the value of the system overhead is independent of the number of executions. The NLDDPG algorithm obtains the optimal action in each execution, that is, the optimal offloading ratio, so that the overall system overhead is minimized. Conversely, random computing fluctuates greatly. Because the offloading ratio of random computing in each execution is uncertain, it is not suitable as an offloading method.

**Figure 8:**
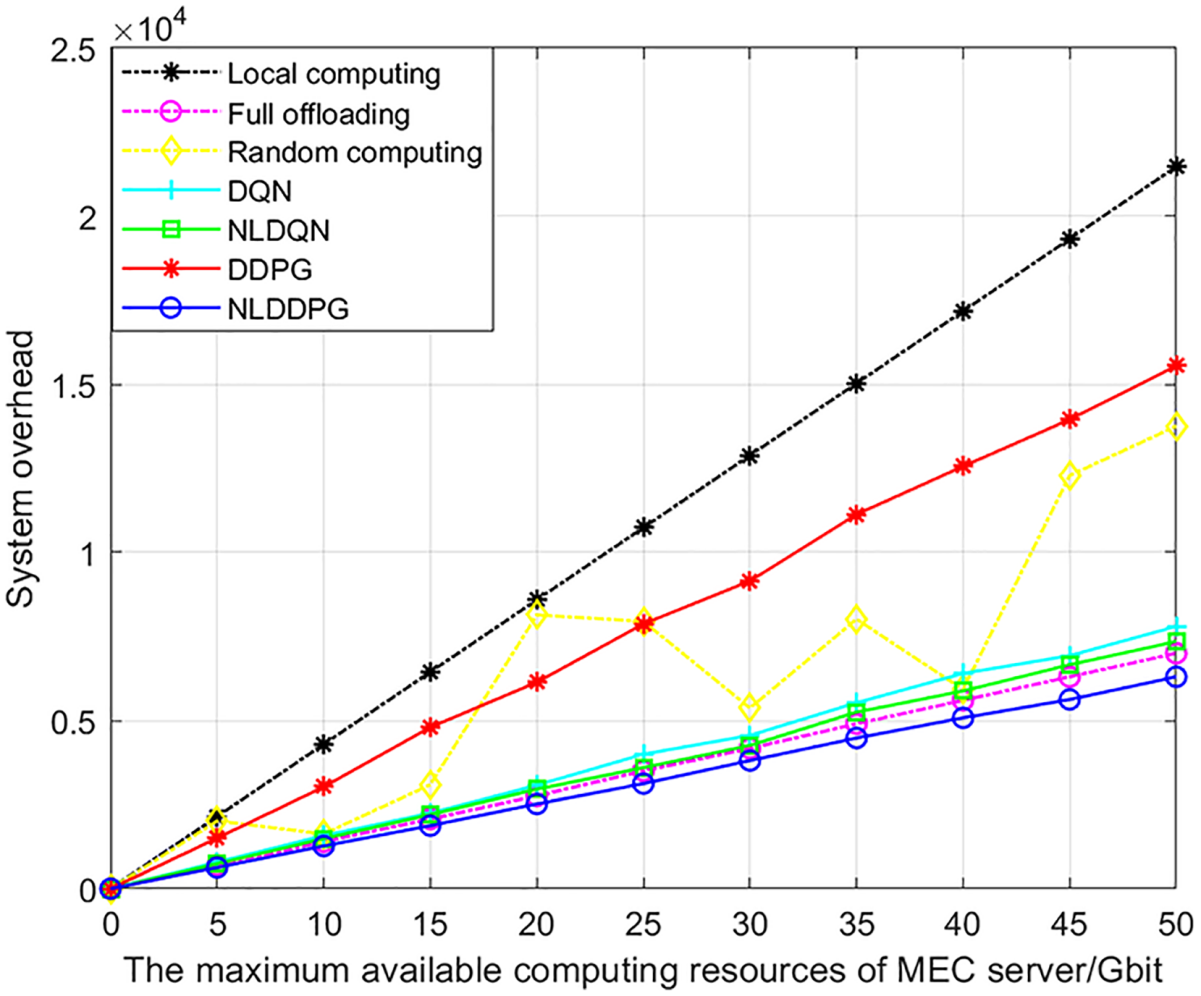
System overhead of different algorithm under different maximum available computing resources of MEC server.

Figure 8 shows the changes of the system overheads of different algorithms with the increasing maximum available computing resources of MEC server. With the increase of the maximum available computing resources, the system overheads of all algorithms increase. This is because with the increase of the maximum available computing resources of the MEC server, the number of vehicle tasks that the system can calculate also increases, so the overall system overhead increases. The rate of increase for the other algorithms is approximately constant, except that random computing has an indeterminate proportion of offloading. This is because the system overhead is positively related to the maximum available computing resources. To sum up, the NLDDPG algorithm has a significantly lower system overhead than the other offloading methods, and the advantage increases as the maximum computation increases.

**Figure 9:**
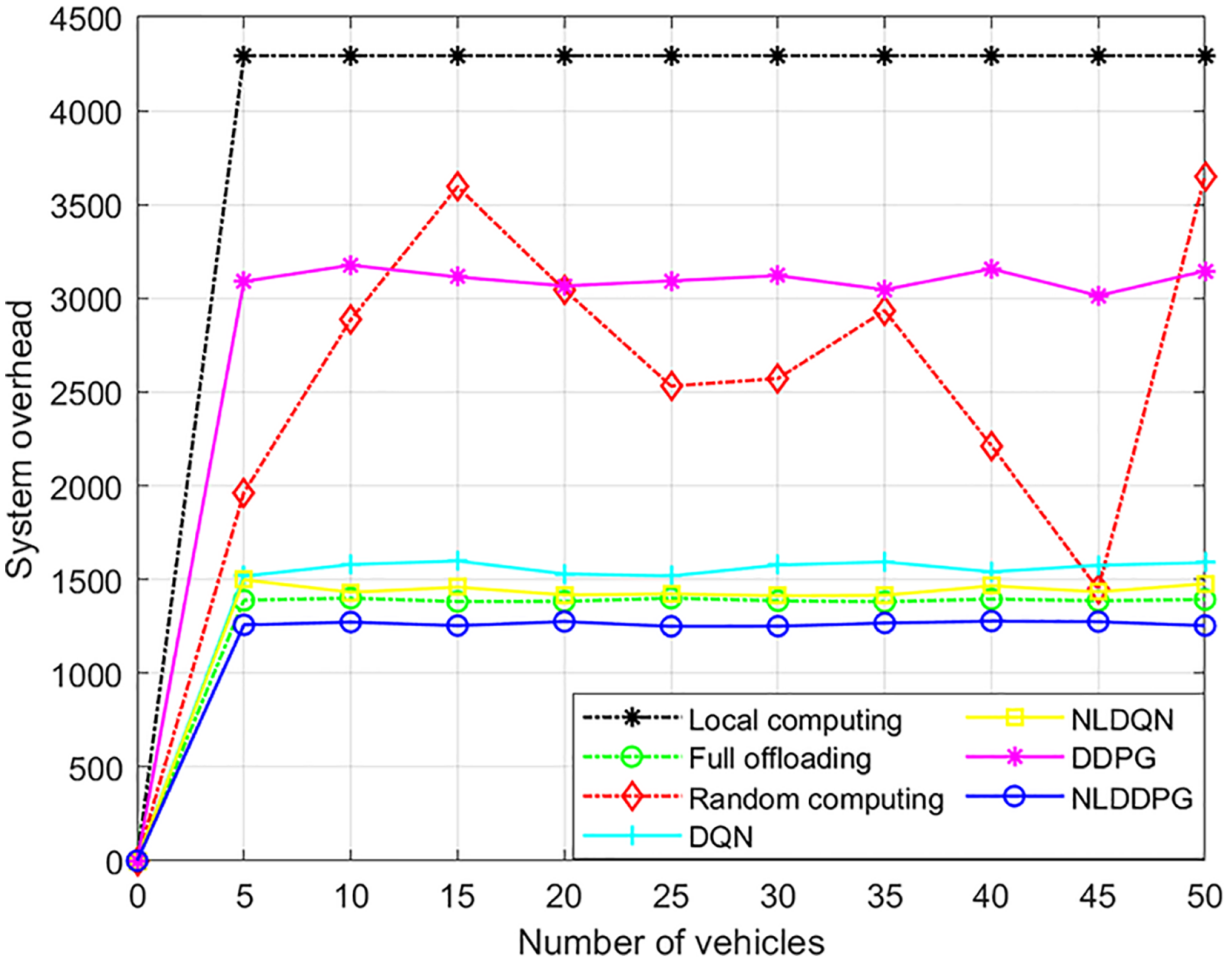
System overhead of different algorithm under different vehicles.

Figure 9 shows the changes of the system overheads of different algorithms with the increasing vehicles. The system overhead changes only slightly as the number of vehicles increases, because the system overhead is closely related to the maximum computation of the MEC server and not to the number of vehicles. As the number of vehicles increases, the number of tasks increases, but the number of tasks that can be calculated has an upper bound, which is limited by the maximum available computing resources of the MEC server. Therefore, the maximum available computing resources remain unchanged, the system overhead will not change significantly. Moreover, it can be concluded from the Fig. 9 that the system overhead of the NLDDPG algorithm is much smaller than that of the local computing, full offloading and random computing for the NLDDPG algorithm obtains the optimal offloading ratio.

## Conclusion

The task offloading decision for vehicular edge computing is one of the key technologies to improve the service capabilities of IoV. The main goal of the proposed task offloading decision algorithm is to be able to flexibly select the edge computing mode based on the task characteristics and network status. Different from the distributed decisions by different independent vehicles in existing work, this article proposes a central vehicular edge computing task decision algorithm based on deep reinforcement learning. Currently, deep reinforcement learning techniques are mostly used to solve discrete actions (local computation and full offloading), such as DQN, in the field of offloading decisions for connected vehicle edge computing tasks. however, the algorithm proposed in this article can solve high dimensional continuous actions (partial offloading). Based on the task characteristics and network status, MEC server can make an optimal offloading decision for minimizing system overhead. The deep reinforcement learning method is used to improve the effectiveness and accuracy of decision. Experimental results illustrate that the decision results of NLDDPG algorithm proposed in this article can effectively reduce system overhead. At present, we only consider the multi-task decision problem within the coverage of a single MEC server, so the decision results still have a certain degree of locality. In the next step, we will study the task offloading decision problem in a multi-MEC server multi-task request environment and use the collaboration mechanism to construct a hierarchical hybrid decision strategy.

## Additional Information and Declarations

### Data Availability

The following information was supplied regarding data availability: The code is available at Github: https://github.com/YYYYYYHuang/PeerJ-Computer-Science.git.
